# Predictors of Delayed Recovery in Ambulatory Advanced Endoscopic Procedures

**DOI:** 10.1002/deo2.70200

**Published:** 2025-09-08

**Authors:** Zoilo K. Suarez, Alexandria Lenyo, Philip M. Frasse, Derek J. Platt, Thomas Hollander, Talwinder Nagi, Michael DeDonno, Vladimir Kushnir, Juan Reyes Genere

**Affiliations:** ^1^ Internal Medicine Department University of Arizona College of Medicine Phoenix Arizona USA; ^2^ Internal Medicine Department Florida Atlantic University Charles E. Schmidt College of Medicine Boca Raton Florida USA; ^3^ Internal Medicine Department Vanderbilt University Medical Center Nashville Tamil Nadu USA; ^4^ Division of Gastroenterology Washington University School of Medicine St. Louis Missouri USA; ^5^ Department of Gastroenterology Geisinger Medical Center Danville Pennsylvania USA

**Keywords:** advanced endoscopy, adverse events, complex endoscopy, length of stay, recovery time

## Abstract

**Background:**

There is a scarcity of data evaluating patient‐related outcomes of advanced or complex endoscopy (ACE) through the post‐anesthesia recovery course. Yet, gastrointestinal distress following ACE can occur commonly, and this may impact recovery length of stay and the approach to post‐anesthesia care. This study aimed to describe post‐procedural symptoms in patients undergoing ACE and identify factors that influence recovery time and course.

**Methods:**

We retrospectively reviewed a prospectively collected database of patients who underwent ambulatory ACE. Procedural‐related symptoms, recovery time, dismissal rate, and adverse events (AE) were recorded. Factors associated with extended recovery time were analyzed using multiple regression analysis. Secondary outcomes included unplanned hospitalization rate and AEs.

**Results:**

A total of 172 patients were included, with a mean age of 62.77 years (standard deviation 14.176). 64 patients (37.2%) had an extended recovery time. Abdominal pain and nausea were the most common symptoms associated with extended recovery. Female sex, longer procedure duration, and higher post‐procedural pain scores at 30 min were significantly associated with extended recovery (*p* < 0.05). The procedural‐related AE rate was 5.8%, and the overall unplanned hospitalization rate was 3.5%.

**Conclusions:**

Procedural‐related symptoms following ACE are common, and female sex, duration of procedure, and post‐procedural pain score at 30 min are predictive of length of stay. These data provide insight into optimizing the approach to post‐procedure care in ACE.

## 1 Introduction

Advanced and complex endoscopy (ACE) plays a vital role in diagnosing and treating many gastrointestinal disorders. While these procedures are oftentimes ambulatory and minimally invasive, they are not without risks for procedural‐related pain, gastrointestinal distress, or adverse events (AEs). However, there is a paucity of studies evaluating how these factors may impact post‐anesthesia care unit (PACU) length of stay, which, in turn, may influence strategies for optimizing delivery of care, resource allocation, as well as personal and health system burdens. Prior studies have investigated recovery outcomes for patients undergoing standard gastrointestinal endoscopy and have found that age, female sex, along with type and dosage of anesthetic administered are risk factors for prolonged recovery time [1, 2]. Corroborating studies evaluating ACE are lacking, and it remains unclear if pre‐emptive measures to enhance endoscopic recovery are indicated in this population outside of bariatric endoscopy [3]. Yet, since these procedures are lengthier and often associated with procedural‐related symptoms, formalizing outcomes in this area is needed. In this study, we evaluated the post‐anesthesia care of patients presenting to an ambulatory endoscopy center for ACE. We aimed to describe the general profile of procedural‐related symptoms and identify factors that negatively impact post‐ACE recovery time. Secondarily, we aimed to observe the rate of unplanned hospital admissions and AE.

## 2 Methods

We retrospectively reviewed a prospectively collected database recording observations after outpatient ACE between January and April of 2022. Washington University Institutional Review Board approval was obtained under the identification number 202009124. Inclusion criteria for the study included the following: [1] patients over the age of 18 and [2] undergoing any of the following procedures: endoscopic retrograde cholangiopancreatography (ERCP), endoscopic ultrasound (EUS) with needle sampling or therapeutic intent, upper or lower advanced endoscopic tissue resection (including complex endoscopic mucosal resection), device‐assisted enteroscopy with resection or hemostatic therapy, radiofrequency ablation, luminal stent placement, variceal banding, balloon dilation, percutaneous gastrostomy tube placement, or endoscopic sleeve gastroplasty. Patients with prior complications and incomplete records were excluded. Procedures were performed by one of seven endoscopists, all of whom had undergone formal training in advanced endoscopic techniques. All endoscopic procedures were performed utilizing carbon dioxide (CO2) insufflation under monitored anesthesia support within a tertiary academic medical center. The indication for administering deep sedation versus general anesthesia was decided at the discretion of the staff anesthesiologist and/or gastroenterologist on a case‐by‐case basis. Our unit uses propofol‐based anesthesia either alone or in combination with a benzodiazepine or opiate. Following ACE, patients were moved to the PACU for monitoring until they met criteria for dismissal by standard Aldrete scoring >8. Post‐procedure hospital admissions were recorded and classified as planned or unplanned, depending on whether the decision was made before or as a result of events during or after the procedure.

### 2.1 Study Variables and Assessments

Baseline data collected included age, sex, Beck's Anxiety Inventory (BAI), and Rhode's Index of Nausea, Vomiting, and Retching (RINVR). Pre‐endoscopy pain, nausea, and bloating scores measured by the Likert scale (ranging 0–5) were also obtained to permit symptom comparison post‐ACE. Medical records were systematically reviewed to collect procedure times, defined as the duration from the administration of sedation to the removal of the endoscope, and anesthesia medication records. Patients were then assessed in the PACU and asked to report pain, nausea, and bloating scores every 15 min until dismissed home or admitted to the hospital. Episodes of vomiting were recorded throughout these intervals. The need for medical intervention to treat symptoms, total recovery time, final disposition, and AE were recorded. To capture delayed AE or unplanned hospital admissions, patients were contacted via telephone within 8–15 days to inquire about their health status.

### 2.2 Outcomes

The primary outcome was to determine the overall rate of procedural‐related symptoms and extended recovery. The latter was defined as greater than 60 min in the PACU, and this was chosen given that post‐ACE monitoring in our unit ranges from 30 to 60 min, and in alignment with similar literature [4, 5]. Greater lengths of stay were considered extended. We also sought to run multiple regression analysis to determine factors associated with extended recovery, given that the sample size was insufficient for propensity score matching. The analysis was structured into four distinct blocks. The first block examined demographic factors, including age and sex. The second block introduced pre‐procedural variables, including the BAI and the RINVR score. The third block evaluated the duration of the procedure, while the final block assessed the post‐pain score at 30 min. Secondary outcomes of interest were the rate of unplanned hospital admissions and the AE rate.

### 2.3 Statistical Analysis

Continuous variables were presented as mean ± standard deviation (SD) or median and interquartile range (IQR). A T‐test was used to make comparisons of continuous values. Categorical variables were reported as counts and frequencies, and these were analyzed using chi‐squared and Fisher's exact test, when indicated. A hierarchical regression analysis, analysis of variance, and multiple regression analysis were performed using the software STATA, version 17.0 (StataCorp, USA). A p‐value <0.05 was considered statistically significant.

## 3 Results

A total of 172 patients were included in the dataset. Demographic, medical history, and baseline data are summarized in Table 1. There were 89 females and 83 males, average age was 62.77 years (SD = 14.176). The average pre‐procedure BAI score was 2.87 (SD = 4.062), and the RINVR score was 1.40 (SD = 3.75). The procedural data and AE are in Table 2. ERCP was the most common type of procedure (*n* = 81), followed by EUS (*n* = 56). The average duration of procedures was 25.35 min (SD = 22.714). Most procedures were performed using deep sedation with monitored anesthesia care and balanced propofol (n = 163, 85.7%), while the remaining were done with general anesthesia. There were 18 AE recorded, 3 related to intraprocedural bleeding that required directed endoscopic intervention, and this was successfully managed in all cases. The remaining 15 occurred in the post‐procedure phase, 1 requiring unplanned hospitalization at the same endoscopy encounter and 5 requiring unplanned hospitalization following dismissal home. This amounted to an overall unplanned hospitalization rate of 3.5%. Over 30% (5/15) of these delayed AEs were not directly related to endoscopy and included an acute stroke, hypertensive urgency, unspecified corporal swelling, and two mechanical falls.

**TABLE 1:** Demographics and baseline symptom scores.

Variable (Total *n* = 172)		
Mean age (± standard deviation)	62.77	±14.17
Sex (n, %)		
Female	89	51.7%
Male	83	48.3%
Pertinent Past Medical History (n, %)		
Diabetes Mellitus	48	27.9%
Opiate dependent chronic pain	10	5.8%
Major depressive disorder	36	20.9%
General anxiety disorder	31	18.0%
Functional gastrointestinal disorders	3	1.7%
Baseline symptom assessments (n, %)		
Beck's Anxiety Inventory Score		
Minimal (0–7)	157	91.3%
Mild (8–15)	12	7.0%
Moderate (16–25)	3	1.7%
Severe (26–63)	0	0%
Rhodes index of Nausea, Vomiting, and Retching score		
Mild (1–8)	164	95.3%
Moderate (9–16)	6	3.5%
Severe (17–24)	1	0.6%
Great (25–32)	1	0.6%
Pain score		
No pain	129	75%
Mild pain (1–3)	16	9.3%
Moderate pain (4–7)	24	14.0%
Severe pain (8–10)	3	1.7%

**TABLE 2:** Summary of endoscopic procedures and safety outcomes.

Procedure (Total *n* = 172)	*n*	General anesthesia (*n*, %)	Deep sedation (*n*, %)	Mean Duration of procedure, mins (SD)
Endoscopic retrograde cholangiopancreatography	81	15 (18.5%)	66 (81.5%)	28.8 (28.78)
Endoscopic ultrasound	56	6 (10.7%)	50 (89.3%)	25 (13.82)
Single balloon enteroscopy	1	0 (0%)	1 (100)	51 (N/A)
Endoscopic resection	12	1 (8.3%)	11 (91.7%)	41.1 (14.44)
Esophageal stenting	1	0 (0%)	1 (100%)	8 (N/A)
Endoscopic dilation	24	1 (0.4%)	23 (99.6%)	15.5 (13.02)
Esophageal varices banding	4	0 (0%)	4 (100%)	7.8 (1.89)
Endoscopic sleeve gastroplasty	1	0 (0%)	1 (100%)	40 (N/A)
Percutaneous endoscopic gastrostomy	4	3 (75%)	1 (25%)	38 (11.40)
Endoscopic cystogastrostomy	1	1 (100%)	0 (0%)	11 (N/A)
Esophageal radiofrequency ablation	6	0 (0%)	6 (100%)	13.7 (7.63)
Summary of adverse events				
Intraprocedural adverse events (*n* = 3)			Postprocedural adverse events (*n* = 15)	
Gastrointestinal bleeding	3 (100%)		Gastrointestinal Bleeding	1 (7%)
			Infection	1 (7%)
			Perforation	1 (7%)
			Acute pain	3 (20%)
			Acute nausea/ vomiting	2 (12%)
			Dysphagia	1 (7%)
			Acute Pancreatitis	1 (7%)
			Non‐procedure related	5 (33%)

Abbreviation: N/A, not applicable.

The average time in recovery was 68.07 min (SD = 31.657), with a range from 30 to 225 min. The total number of patients that required greater than 60 min of recovery was 64 (37.2%), and the mean duration of extended recovery was 104 min (SD 3.5), as compared to an average of 52 min (SD 2.7) in the expected recovery group. Table 3 summarizes the reasons for extended recovery. Abdominal pain (47%) and nausea (20%) were the most common symptoms causing extended recovery, with vomiting and bloating amounting to a small sum (3% each). There were 17 (27%) patients who had extended recovery times without bothersome symptoms. Out of these patients, a high number (17.7%) were eventually admitted to the hospital, and the remaining were dismissed home, perhaps indicating the primary factor for prolonged observation was safety after a complex procedure. A comparison of variables between those with extended recovery versus expected recovery times is in Table 4. Females (*p* < 0.001), longer procedure times (16 vs. 22.5 min, *p* = 0.018), and patients who underwent ERCP with biliary sphincterotomy (*p* = 0.023) or pancreatic intervention (*p* = 0.023) were associated with a longer recovery. In terms of symptoms, abdominal pain (*p* = <0.001) and nausea (*p* = 0.002) were associated with prolonged recovery. However, there were no differences in recovery time between patients who received deep sedation versus general anesthesia. Also, there was no statistically significant association between anesthetic dosages and recovery time (Table 5) (F(3, 167) = 1.82, *p* = 0.145).

**TABLE 3:** Summary of post‐advanced and complex endoscopy recovery.

Variable (Total *n* = 172)	
Expected recovery (*n*, %)	108 (62.8%)
Extended recovery (*n*, %)	64 (37.2%)
Reasons for extended recovery (*n*, %)	
Pain	30 (47%)
Nausea	13 (20%)
Vomiting	2 (3%)
Bloating	2 (3%)
Non‐medically related	17 (27%)

**TABLE 4:** Factors Associated with Extended Versus Expected Recovery.

Variable	Expected Recovery Group^1^ *n* = 108	Extended Recovery Group^2^ *n* = 64	*p*‐Value
Mean duration of recovery, minutes (± SD)	52 (2.7)	104 (3.5)	<0.001
Mean age (± SD)	62.71 (14.2)	62.88 (14.13)	0.940
Female (*n*, %)	42 (39%)	47 (73%)	<0.001
Male (*n*, %)	66 (61%)	17 (27%)	
Baseline BAI Score			
No to minimal anxiety	101 (93%)	56 (88%)	0.260*
Mild to Moderate Anxiety	7 (7%)	8 (12%)	
Baseline RINVR			
None–Mild	105 (97%)	59 (92%)	0.150*
Moderate–Great	3 (3%)	5 (8%)	
Procedure			
Median duration of procedure (minutes), IQR	16 (20)	22.5 (24.75)	0.018
Endoscopic retrograde cholangiopancreatography	42 (39%)	39 (61%)	0.005
Including biliary sphincterotomy	6 (5%)	10 (16%)	0.028
Excluding biliary sphincterotomy	33 (31%)	29 (45%)	0.051
For pancreatic intervention	9 (8%)	13 (20%)	0.023
Endoscopic ultrasound	35 (32%)	21 (33%)	0.217
Single balloon enteroscopy	0 (0%)	1 (2%)	0.372*
Endoscopic resection	8 (7.4%	4 (6.2%	1.000
Esophageal stenting	0 (%)	1 (1.5%)	0.372*
Endoscopic dilation	19 (18%)	5 (8%)	0.074
Esophageal varices banding	3 (2.8%)	1 (1.5%)	1.000*
Endoscopic sleeve gastroplasty	0 (0%)	1 (1.5%)	0.372*
Percutaneous endoscopic gastrostomy	2 (1.8%)	2 (3%)	0.629*
Cystogastrostomy	1 (1%)	0 (0%)	1.000*
Esophageal radiofrequency ablation	5 (4.6%)	1 (1.5%)	0.414*
Post‐procedural symptoms requiring medical intervention			
Nausea	4 (3.7%)	13 (20%)	<0.001*
Vomiting	0 (0%)	2 (3.2%)	0.138*
Bloating	1 (0.9%)	0 (0%)	1.000*
Pain	13 (12%)	20 (31%)	0.002

^1^Expected Recovery was defined as ≤ 60 min.

^2^Extended Recovery was defined as > 60 min.

**TABLE 5:** Multiple regression results for the influence of anesthetic doses and recovery time.

Recovery time	B	95% CI for B		SE B	Β	R^2^	ΔR^2^
		LL	UL				
Model						.032	.014
Constant	63.931^***^	51.488	76.374	6.303^***^			
Fentanyl dose	.104	−.006	.213	.055	.144		
Propofol dose	.006	−0.24	.036	.015	.028		
Midazolam dose	4.997	−3.662	13.655	4.386	.087		

Abbreviations: B, unstandardized regression coefficient; CI, confidence interval; LL, lower limit; UL, upper limit; SE β, standard error of the coefficient; β, standardized coefficient; R^2^, coefficient of determination; ΔR^2^, adjusted R^2^.

* *p* < 0.05.

** *p* < 0.010.

*** *p* < 0.001.

**FIGURE 1:**
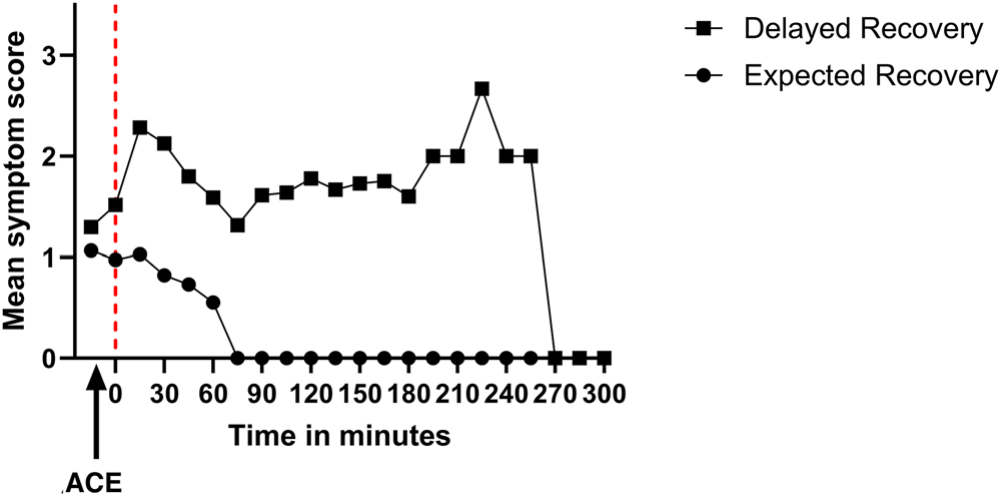
Comparison of average post‐ACE pain scores based on recovery.

Relative to the inferential analysis, 158 patients were included, as 14 were missing pertinent data. A hierarchical regression analysis was performed to evaluate factors associated with extended recovery. The first model included age and sex statistically significantly predicted total time in recovery, F(2, 155) = 5.832, *p* < 0.05. The adjusted R2 for the first model was.058. The second model, which included age, sex, BAI score, and RINVR score, statistically significantly predicted total time in recovery, F(4, 153) = 3.121, *p* < 0.05. The third model, which included age, sex, BAI score, RINVR score, and duration of procedure, statistically significantly predicted total time in recovery, F(5, 152) = 4.493, *p* < 0.001. The addition of the procedure's duration led to a statistically significant increase in the adjusted R^2^ of 0.049, while the overall model increased to 0.100. The final model, which included age, sex, BAI score, RINVR score, duration of the procedure, and post‐procedural pain score at 30‐min statistically significantly predicted total time in recovery, F(6, 151) = 5.689, *p* < 0.001. The addition of the post‐procedural pain score at 30 min led to a statistically significant increase in the adjusted R^2^ of 0.052, while the final model increased to.152. Based on the overall model, only sex, duration of procedure, and post‐procedural pain score at 30 min collectively predicted 15.2% of the variance in total time in recovery (Table 6). As a result, the following regression equation was developed to predict total recovery time. Total Time in Recovery = 36.624 + (12.758 × Sex) + (0.274 × Duration of Procedure) + (3.459 × post‐procedural pain score at 30 min). Figure 1 shows a graphical illustration of pain scores pre‐ and post‐ACE comparing patients with extended and expected recovery times.

**TABLE 6:** Hierarchical multiple regression predicting total time in recovery.

	Model 1		Model 2		Model 3		Model 4	
Variable	B	β	B	β	B	β	B	β
Constant	43.219^**^		44.595^**^		37.164^*^		36.624^**^	
Age	−0.004	−0.002	−0.021	−0.009	−0.010	−0.005	0.011	0.005
Sex	16.675^***^	0.264	15.570^**^	0.247	14.677^**^	0.233	12.758^*^	0.202
BAI Score			0.671	0.082	0.709	0.087	0.424	0.052
RINVR Score			−0.381	−0.043	−0.473	−0.053	−0.394	−0.044
Duration of Procedure					0.336^**^	0.110	0.274^*^	0.189
Post‐procedural pain score at 30 min							3.459^**^	0.246
R^2^	0.070		0.075		0.129		0.184	
F	5.832^**^		3.121^*^		4.493^***^		5.689^***^	
∆R^2^	0.058		0.061		0.100		0.152	

*N* = 158 (patients with all required data points).

* *p* < 0.05.

** *p* < 0.010.

*** *p* < 0.001.

## 4 Discussion

The present study aimed to investigate patient recovery following ACE and identify important factors that influence the recovery course. While most patients may be dismissed from the PACU within a 60‐minute timeframe, about one‐third require an extended period of observation, averaging 104 min in duration. Procedural‐related pain or nausea was primarily responsible for extended recovery. Regression analysis revealed that factors predictive of a greater recovery time were female sex, longer procedural duration, and post‐procedural pain score at 30 min.

Most of the literature reporting on endoscopy recovery has studied standard upper endoscopy and colonoscopy. A prior report has shown prolonged recovery is associated with factors relative to anesthesia recovery, including age (odds ratio [OR] 1.025), female sex (OR 1.657), and intraprocedural midazolam dosage (OR 1.019) [1]. Other investigations on post‐endoscopy recovery time have also supported the idea that the length of stay is mediated by the number of sedatives dosed and the patient's alertness upon arrival to PACU [2]. Corroborating evidence has been documented as well with reduced recovery times using a short‐acting propofol‐based anesthetic, as compared to benzodiazepine and opiate‐based sedation [6, 7]. This data has led to widespread practice change with many modern endoscopy centers preferring propofol‐based sedation. However, we cannot extrapolate the findings from studies evaluating standard endoscopic procedures into ACE for a few reasons. First, the post‐procedural period is categorically different, as recovery times are generally longer and outside of the expected window of anesthesia recovery. In our unit, upper endoscopy and colonoscopy typically require a 20‐min length of stay before dismissal. As opposed to patients who underwent ACE, who are generally monitored for 30–60 min. Second, it is common that ACE is performed under propofol‐based sedation, and this may result in reduced anesthesia recovery time, as compared to moderate sedation strategies. Our data support this, as the type of anesthesia or agents given did not influence recovery time.

Procedural‐related symptoms are suspected to be a primary determinant of ACE recovery time. Many studies have described post‐procedure related pain or discomfort for endoscopic interventions ranging from 1% to 51.4%, but the minority focused on evaluating symptoms beyond pain or relative to recovery length of stay [8, 9, 10, 11, 12, 13, 14, 15, 16, 17]. One study retrospectively reviewed 336 patients who underwent colonic endoscopic mucosal resection. In this cohort, 19.9% of patients had abdominal pain following the procedure, of which 29.9% required extended recovery time, defined as a length of stay >2 h, and 23% needed treatment with an opiate analgesic. Most patients were dismissed home, and 6 required unplanned hospitalization [18]. A randomized control study that also assessed patients undergoing colonic endoscopic mucosal resection evaluated post‐procedural outcomes relative to using a standard injectant or an injectant mixed with epinephrine for submucosal lift [4]. The investigators in this trial found that patients in the control arm required antiemetics and opiates for symptom treatment at a rate of 10% and 20%, respectively, and this increased to 40% and 30% in the epinephrine group, respectively. In turn, the recovery time in the epinephrine group was longer (68 min vs. 52 min), which may have been expected given the higher rate of post‐procedure symptoms. While the findings from these studies suggest an association between post‐procedural symptoms and recovery length of stay, this was not assessed as an outcome. The closest data published in the literature that evaluates the association between post‐procedural symptoms and recovery time comes from a large retrospective study of 31,852 standard colonoscopies, which showed a 6.92 odds ratio for extended recovery in patients with any amount of post‐procedural pain [19].

Our cohort of patients had a similar rate of 47% post‐ACE abdominal pain and 20% nausea, as compared to the aforementioned studies. Furthermore, we confirmed an association with post‐procedural abdominal pain, nausea, and extended recovery time that was statistically significant. We also evaluated other factors that could be associated with extended recovery, including female sex, ERCP with biliary sphincterotomy and pancreatic intervention, and longer procedural time. However, only female sex, procedural time, and post‐procedural pain at 30 min predicted extended recovery in our regression modeling. These findings were not unexpected. Sex and longer duration of procedure have been shown in other studies to increase the risk of post‐procedural symptoms or recovery times in endoscopy [1, 19, 20]. In fact, the influence of female sex on post‐procedural recovery has been thought to be influenced by an increased susceptibility to nausea, as well as differences in visceral nociceptive pathways, drug metabolism, hormones, and hypervigilance [16, 21].

The more intriguing finding is relative to the post‐procedure abdominal pain score at 30 min, as it may serve as a predictor for recovery time. This observation suggests there could be an opportunity for early intervention to facilitate recovery length of stay in patients undergoing ACE.

ACE also exhibited a favorable safety profile. The overall AE rate was 10.4%, with a delayed AE rate of 8.7%. All three intraprocedural events were due to bleeding and successfully managed endoscopically, while 33% of delayed AEs were not directly related to the endoscopic procedure. This leaves an endoscopic‐related delayed AE rate of 5.8%, which is similar to prior reports on ACE safety overall [22, 23, 24, 25]. Finally, hospitalization rates were low at the index encounter, with an overall rate of 5.2% and an unplanned rate of 0.6%. Additionally, the post‐dismissal hospitalization rate was 2.9%, which yields an overall unplanned hospitalization rate of 3.5% in our cohort. Given the low number of events, further analysis identifying risk factors associated with unplanned hospitalizations could not be elucidated.

Our study had limitations to consider. First, our sample size was relatively small with respect to some procedures represented, and this may have hidden the effects of interested outcomes. Partly this is because we included a broad definition of ACE encounters for the study. However, it also provided a sample population representative of a typical contemporary advanced endoscopy practice, and there was sufficient data to evaluate the primary endpoint. Another consideration stems from the nature of a post‐hoc analysis, which carries an inherent risk of type 1 error due to sampling bias. However, we mitigated this by first enrolling patients on an incoming basis, including a wide range of ACE, and applying regression analysis to eliminate confounders. Other considerations are regarding generalizability. Functional bowel disorders were not well represented in our cohort, and this may influence the prevalence of post‐procedural symptoms. Our study was also conducted at a single tertiary center, and these findings may not be generalizable to all endoscopy centers practicing ACE.

Despite these limitations, our findings provide important insight into understanding patient recovery following ACE. Our data support the notion that recovery time after ACE is largely mediated by post‐procedural symptoms, as opposed to factors related to anesthesia practice. Endoscopists may consider the patient's sex or long duration of procedure as criteria for initiating a proactive placement of as‐needed orders for nausea and pain following procedures. The endoscopist may also strategically plan patient assessments prior to the 30‐min period, thereby allowing early intervention in the course of patient recovery to manage pain. Nursing workflow optimizations can be run in parallel for close patient symptom surveillance and facilitate prompt communication with the treating endoscopist, if issues were to arise.

## 5 Conclusion

In conclusion, procedural‐related symptoms following therapeutic endoscopic procedures are common, and female sex, duration of procedure, and post‐procedural pain score at 30 min are predictive of length of stay in the recovery unit. These data may be informative for optimizing endoscopy unit operations and post‐anesthesia care. To confirm our findings, further research involving a larger sample and a longer study period would be recommended.

## Author Contributions

**Zoilo K. Suarez**: Data abstraction, analysis, original draft preparation, and final edits. **Alexandria Lenyo**: Data curation. **Phillip Frasse and Derek Platt**: Data curation. **Thomas Hollander**: Data curation; methodology. **Talwinder Nagi**: Writing ‐ original draft. **Michael DeDonno**: Formal analysis; methodology; writing ‐ original draft. **Vladimir Kushnir**: Writing ‐ review and editing. **Juan Reyes Genere**: Conceptualization; data curation; formal analysis; methodology; project administration; resources; software; supervision; validation; writing ‐ review and editing.

## Ethics Statement

The Washington University Institutional Review Board approved this study under the identification number 202009124.

## Consent

Informed consent was obtained from all patients before data collection.

## Conflicts of Interest

Vladimir Kushnir is a consultant for Boston Scientific. Juan Reyes Genere is a consultant for Boston Scientific and Edulis Therapeutics Inc. All other authors declare no conflicts of interest.

## Clinical Trial Registration

N/A.
